# Best practices for engaging lived experience experts in dialogues about climate change and mental health: lessons learned from a global effort

**DOI:** 10.1186/s44263-026-00321-7

**Published:** 2026-10-13

**Authors:** Britt Wray, Ans Vercammen, Lian Zeitz, Sacha Wright, Vashti-Eve Burrows, Jeannie Yamazaki, Suhailah Jasmin Ali, Kyle Hill, Jemaima Tiatia-Siau, Kezang Wangmo, John Jamir Benzon Aruta, Gail Co, Renzo R. Guinto, Emma L. Lawrance

**Affiliations:** 1 Department of Psychiatry and Behavioral Sciences, Stanford School of Medicine, Stanford University, Stanford, CA USA; 2 School of Population Health, Curtin University, Perth, Australia; 3 Collective Change Lab, Lincoln, MA USA; 4 Force of Nature, London, UK; 5 London School of Hygiene and Tropical Medicine, London, UK; 6 School of Clinical Medicine and Research, The University of the West Indies, Nassau, Bahamas; 7 Department of Educational and Counseling Psychology, University at Albany, State University of New York, Albany, NY USA; 8 Jubilee Australia Research Centre, Sydney, Australia; 9 Division of Environmental Health Sciences, School of Public Health, University of Minnesota, Minneapolis, MN USA; 10 School of Māori Studies and Pacific Studies, University of Auckland, Auckland, New Zealand; 11 Youth Ambassador, Connecting Climate Minds, Thimphu, Bhutan; 12 Department of Psychology, De La Salle University, Manila, Philippines; 13 Health and Global Policy Institute, Tokyo, Japan; 14 Planetary and Global Health Program, St. Luke’s Medical Center College of Medicine-William H. Quasha Memorial, Quezon City, Philippines; 15 SingHealth Duke-NUS Global Health Institute, Duke-NUS Medical School, National University of Singapore, Singapore, Singapore; 16 Department of Psychiatry, University of Oxford, Oxford, UK; 17 Institute of Global Health Innovation, Imperial College London, London, UK; 18 Grantham Institute for Climate Change and the Environment, Imperial College London, London, UK

**Keywords:** Climate change, Mental health, Lived experience, Justice-oriented research, Community engagement, Community of practice, Regional agenda, Global agenda

## Abstract

The Connecting Climate Minds global initiative sought to advance research and action at the intersection of climate change and mental health by centering the voices of people with lived experience. Through a series of regional and thematic dialogues, Connecting Climate Minds engaged nearly 1,200 participants across seven global regions, including Indigenous communities, smallholder farmers and fisher peoples, and youth. A Lived Experience Working Group guided the integration of lived experience throughout all project components, from co-designing research agendas to producing multimedia storytelling outputs. This perspective reflects on the methodologies, challenges, and lessons learned from Connecting Climate Minds' lived experience engagement, drawing on two global debriefing workshops and extensive project documentation. Key principles that emerged include the importance of appropriately resourcing relationship building ("moving at the speed of trust"), reciprocity, cultural sensitivity, ethical storytelling, and the need for researcher training. The perspective also highlights systemic barriers to meaningful engagement, such as rigid funding structures and institutional resistance to, or inappropriate systems for, power-sharing with impacted communities. By documenting both successes and missteps, this perspective offers practical guidance for researchers, funders, and policymakers committed to inclusive, justice-oriented approaches in climate and mental health research. Ultimately, Connecting Climate Minds demonstrates that centering lived experience not only enriches research quality and relevance but also fosters more equitable and transformative outcomes.

## Background

The Connecting Climate Minds (CCM) project set out to: (1) catalyse a collaborative, transdisciplinary global community of practice and (2) develop prioritised agendas to advance research and action at the intersection of climate change and mental health (see: https://hub.connectingclimateminds.org). From its inception, the project aimed to centre the expertise of people with lived experience of climate-related mental health challenges, incorporating perspectives not traditionally valued or engaged in research efforts. A Lived Experience Working Group (LEWG)–composed of leaders and advocates from community-based non-profits and researchers who have lived experience–was established to guide the integration of lived experience into various aspects of the project. This paper analyzes the processes, outcomes and lessons that emerged from embedding lived experience expertise throughout a global collaborative project (CCM), with the aim of informing future efforts in climate change and mental health research and action.

We report on the outcomes of two post-project online workshops where CCM team members engaged in critical reflection as a methodology [1] to explore our collective experiences of integrating lived experience expertise and engagement into CCM, focusing on what worked well and what did not. The integration of lived experience in CCM builds on the growing literature around community-engaged and co-designed health research that upholds the lived experience of participants as a form of critical expertise that ought to inform research and intervention development from its earliest stages [2, 3]. We hope this perspective will provide valuable insights to address a persistent gap in global climate-mental health research, namely the absence of co-produced, trauma-informed methodologies led by those most affected.

This gap reflects broader histories of extractive and colonial research relationships in which communities, particularly in the Global South and Indigenous communities worldwide, were treated as data sources rather than knowledge holders, contributed to research without direct benefit, and in which the mental health dimensions of environmental injustice have been chronically underfunded and depoliticized. While these issues sometimes stem from behaviour on the part of individual researchers, they are largely systemic issues reflecting the values upholding academic and political structures that determine whose research gets funding, who counts as an expert, who reaps rewards and who takes on the risks. This reasoning behind CCM’s lived experience work supports and advocates for institutional transformation of these structures to hopefully one day arrive at a point where the way in which climate-mental health research is done and who the research benefits matter as much as what research is done, establishing a more epistemically just field.

This paper also seeks to offer practical advice for researchers, funders, policymakers and practitioners who are committed to supporting and empowering lived experience experts in research processes that aim to address climate-mental health challenges.

### The Connecting Climate Minds project

As a global project, CCM was designed to prioritize regional engagement and center regional voices while being closely connected through global coordination [4]. Communities of Practice were established in the seven UN Sustainable Development Goal regions, each coordinated by a Regional Community Convenor (RCC) and with Lived Experience Advisory Groups (LEAGs) and Youth Ambassadors as core members of the Regional Community Teams. To develop a clear, supported and actionable vision, each RCC hosted two multi-stakeholder dialogues, which included contributors with expertise from lived experience. The dialogues resulted in a set of regional agendas, as well as a synthesised global agenda highlighting *research priorities* to understand and address the needs of people experiencing the mental health burdens of the climate crisis, and *opportunities* to translate evidence into policy and practice.

CCM also hosted thematic in-person and online dialogues with members of three disproportionately impacted communities: Indigenous communities, smallholder farmers and fisher peoples, and young people. These communities tend to be underrepresented (or not consulted at all) in decision-making about climate and/or health policies, making their engagement in this process all the more crucial. Finally, CCM conducted a story-gathering process wherein personal narratives of lived experience were recorded for media outreach and to be featured on our global online platform [5]. All project members were oriented in trauma-informed and inclusive practices to create safe spaces for engagement, reflecting CCM’s commitment to addressing the historic legacy of extractive research relationships with communities. A full exploration of the aims and processes of CCM is reported elsewhere [4].

### What is lived experience work and why is it vital?

By lived experience, we mean the unique forms of human-centered knowledge, insight, stories, and expertise that come from a range of personal experiences. In the context of this project, we attend particularly to forms of lived experience that are rooted in and shaped by structural inequity, marginalization, and histories of harm, including those arising from colonialism, displacement, environmental racism, and state violence; the type of extractive practices that cause both climate and mental health harms. We recognize that the term “lived experience” has at times been co-opted to (over)emphasize the perspectives of those with institutional power, which risks diluting its political and epistemic force. Our use of the term in this paper, and in the aims of the work we describe, centres those whose knowledge has been systematically excluded from research and policy processes, time and again. The past decade has seen an increase in the inclusion of lived experience in research on mental health [6]. This growth in inclusive practice marks a departure from previous research practices that often excluded or devalued diverse worldviews and realities [7]. Lived experience inclusion work aims to bring wisdom and expertise from perspectives not traditionally valued or engaged in research efforts and incorporate those insights into a collaborative knowledge generation process. The incorporation of lived experience is meant to transcend the mere dissemination of personal and communal narratives, or superficial inclusion of feedback on projects from lived experience experts who belong to communities the research is meant to serve. Beyond dissemination and feedback lie rich potentials to generate new knowledge and ways of seeing, thinking, and doing led by lived experience when methodologies that were created to push back against historical power imbalances and extractive socio-political dynamics are applied. These include co-design, community-based participatory research, decolonial Indigenous research methodologies, data sovereignty frameworks, collaborative ethnography, standpoint epistemology, and more.

The incorporation of lived experience serves as an integral component of community-based research, addressing gaps in knowledge and unequal power relations across research, policy, narrative development, and practical dialogue-based initiatives [8]. This integration facilitates the introduction of insightful and innovative perspectives rooted in bottom-up first-hand knowledge to the domains of mental health and climate change, helping to ensure that research is conducted in ways that uplift and honour those it aims to serve. At the heart of lived experience work is a recalibration of what it means to be an ‘expert’ in research [9]. Historically, expertise centered the background of the researcher, often rooted in academic credentials and professional achievement. While this form of expertise remains crucial to effective research, narrow views of whose voices matter most diminish the potential for the invaluable wisdom that comes from personal experience to guide research processes, often resulting in outputs that do not reflect the needs of those most impacted [10]. The implications of this gap are historically evident in research where traditional ‘experts’, often unintentionally, perpetuate harm and overlook insights necessary for a comprehensive understanding of challenges and solutions [11]. These harms are well documented across many fields and have often included extracting lived experience as data to generate academic capital that benefits the researcher’s career without mutual agreement and consent (i.e. parachute research [12]), or without reciprocity such as fair compensation or acknowledgement/authorship [13]; operating only within institutional/Western frameworks that structurally devalue the knowledges, concepts, and languages that lie at the heart of a phenomenon being studied [14, 15]; pathologizing normal experiences that are germane to the community [16]; misrepresenting and misinterpreting insider community knowledge [17, 18]; retraumatizing individuals who have been previously harmed through insensitive questioning [19]; violating cultural traditions (such as publishing about sacred knowledges or rituals that have been observed), and raising expectations for long term support that are not met, among others [20].

This reframing requires role clarity, fair compensation, and decision-making power for lived experience contributors. These requirements were possible to meet in CCM due to the way the project was structured to allow for flexibility, transparency, and sensitivity to power dynamics that might require emergent and caring responsiveness at any moment, making it an exemplary project for lived experience integration.

### Positionality of the authors

The co-authors of this paper occupy diverse and sometimes contradictory positions in relation to the communities whose voices CCM sought to centre. We include community organizers and advocates with deep ties to affected communities, researchers and practitioners from regions directly experiencing the mental health burdens of climate change, and researchers housed at major Western academic institutions. Some of us hold lived experience of the climate mental health challenges described in this paper; others hold institutional power that has historically been complicit in the extractive research practices we critique; while others hold both or neither simultaneously. Some of us are global majority members from the Global South, some of us are Indigenous, while others are settlers whose ancestors propagated and/or benefitted from colonization. This heterogeneity shaped both the project’s strengths and its limitations. We acknowledge that working within institutional structures (including those of the funder and host universities) constrained our ability to enact the full transformative potential we aspired to. We approach this paper as an evolving practice with opportunity for growth rather than a completed account.

### Engaging with lived experience experts throughout the project

Lived experience was carefully integrated throughout all aspects of CCM. Below is a description of how lived experience was integrated across both the process and outcomes of CCM. A description of the overall CCM project is available elsewhere [4] and two papers are forthcoming on the project methods and lessons learned.

### Process

**Fig. 1:**
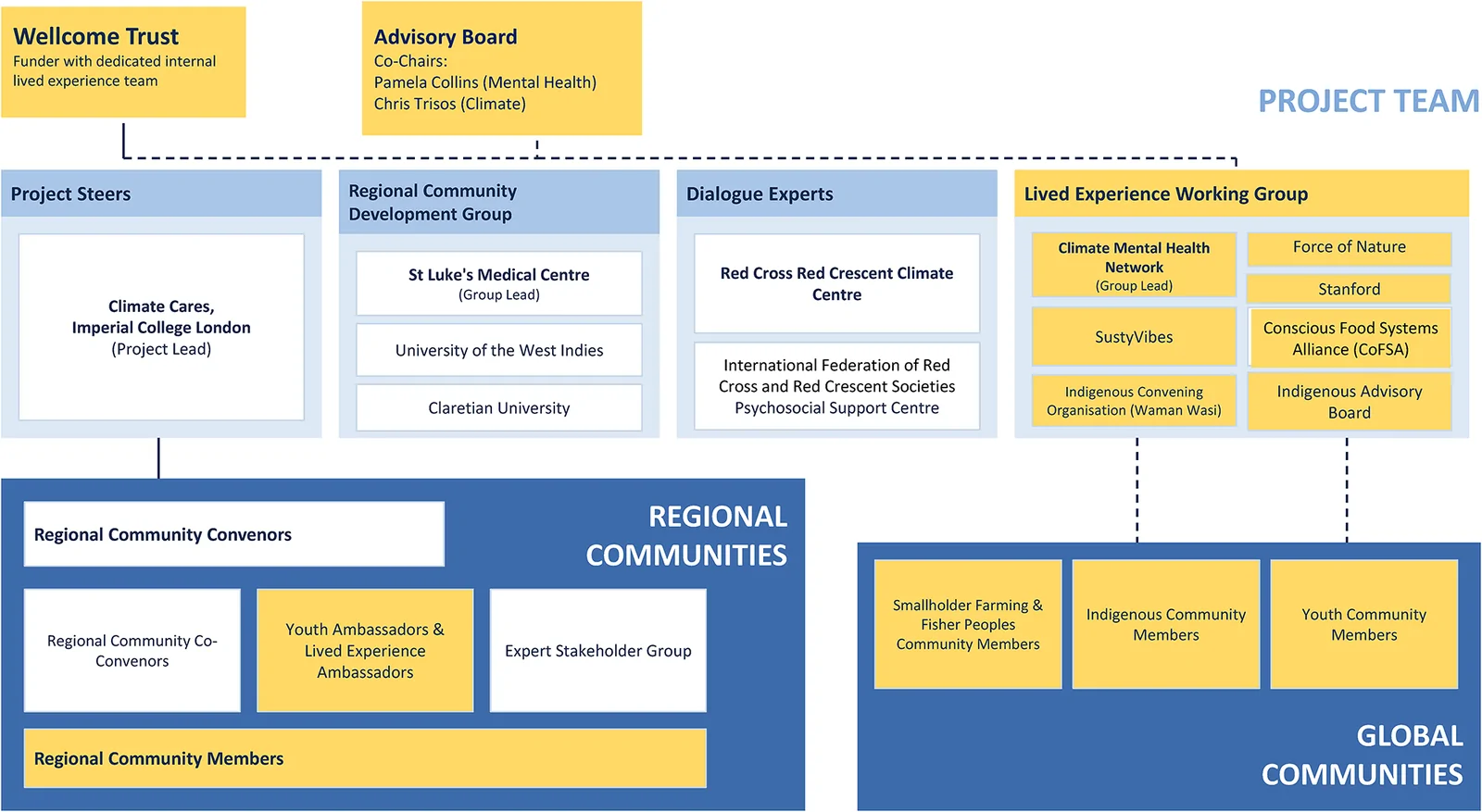
Organizational structure of the Connecting Climate Minds initiative. Areas marked in yellow represent significant involvement of Lived Experience experts or Lived Experience work as the thematic focus of the group.

The project was developed and overseen by the Global Team, which included the LEWG. The LEWG, composed of the non-profit organizations Climate Mental Health Network, Force of Nature, and Sustyvibes alongside Stanford University, ensured that lived experience was fully embedded in all project elements. At a regional level, projects were carried out by Regional Community Teams, led by RCCs and consisting of a LEAG and Youth Ambassadors (YAs). The RCCs were trained and supported by the LEWG in recruiting, convening and working alongside the LEAG and YAs. The LEWG also provided direct support to LEAG and YA members. As part of the Regional Community Teams, the LEAG and YAs supported decision-making and all relevant activities, including recruiting people with relevant lived experiences to be part of dialogues and as storytellers for the lived experience videos (see below), and to gather further stories and insights from their communities to inform the project processes. See Fig. 1 for an overview of the organizational structure of CCM and its participating groups.

Additionally, at a global level, three work streams were led by and for communities with particular sources of impact, vulnerability, and resilience. These were: Indigenous communities, smallholder farmers and fisher peoples, and young people. The LEWG recruited paid advisors, community leaders and organisations from the relevant communities to co-produce these workstreams, embodying the fundamental principle “nothing about us without us” [21]. The opportunity was taken to do tailored and enriched engagement with these three communities beyond what could be achieved through the regional dialogue structures.

### Deliverables

Each Regional Community Team held two three-hour long dialogues online, which brought together lived experience experts, transdisciplinary researchers, as well as practitioners and civil society members. The teams then consolidated the resulting insights into a Regional Research and Action Agenda for climate change and mental health [22].

In March of 2024, a global event was held in Barbados to celebrate the project outcomes and finalise the global research and action agenda. The Global Team, LEWG, RCCs, and select LEAG and YA members attended. The LEWG presented their insights from the project. These insights were summarised in a toolkit entitled *Deepening Research & Practice: A Lived Experience Engagement Toolkit*, which is available on the CCM Global Online Hub for audiences interested in working with lived experience experts in climate change and mental health [23]. The event also included presentations by representatives from the various regional community teams as well as a cultural showcase through which participants shared the vibrancy of their culture and people, including many gifts, traditional foods, clothing, songs and dance.

A series of videos that captured first-person narratives (‘lived experience videos’) were recorded, produced, and disseminated on the CCM global online hub [5] to ensure that the voices of dialogue participants and Regional Community members with lived experience were elevated alongside the research and action agendas. The LEWG led the appropriate guidance for the Regional Communities of Practice to identify, care for, and record the stories of people with lived experiences in inclusive, trauma-informed and non-coercive ways. The lived experience videos were shown at the UN Conference of the Parties (COP) climate negotiations in Dubai known as COP28 to diverse audiences, including on the WHO stage [24]. They proved to be a powerful tool for enabling lived experience experts to share their sources of suffering and resilience with a global and significantly policy-oriented audience.

### Methods of collecting reflections on lived experience inclusion in CCM

Two online workshops were held to record reflections on the successes, challenges and lessons learned from lived experience inclusion in CCM. The workshops combined geographic regions to maximise potential for synchronous discussion across time zones. One was for Europe & North America (ENA), Sub-Saharan Africa (SSA) and Latin America & Caribbean (LAC), while the other was for the Middle East & North Africa (MENA), Central & Southern Asia (CSA), Oceania and Eastern & South Eastern Asia (ESEA). The workshops were facilitated by the RCC and LEWG members who are co-authors of this paper, and participants included members of the LEWG, as well as core members from each RCC who had engaged in lived experience work throughout CCM. Participants who were invited based on the depth of their lived experience engagements were encouraged to invite one additional person with extensive experience in the lived experience aspects of their region’s work throughout the project.

The workshops were 90 min each and held on an online conferencing platform (Zoom, Zoom Communications). They began with a brief check-in, followed by a summary of the workshop’s aims. The remainder was spent generating relevant reflections, captured on the Miro digital whiteboard software (RealtimeBoard, Inc). The Miro board was organised into eight colour-coded categories, and participants were asked to reflect and share their insights by adding digital post-its under each category. The first four categories asked participants to separately describe key lessons for researchers, funders, policy-makers/practitioners, and lessons that might apply to all stakeholders. The remaining categories reflected on successes, opportunities for future learning and improvement, and the sustainability of the lived experience work. We also created a category to capture thoughts not easily classified elsewhere. The Miro boards were circulated to participants before the workshops so they could familiarize themselves with the prompts. Following the workshops, the Miro boards were made available for further comment and for asynchronous contributions from those unable to attend.

The facilitators reviewed and reorganised the raw data by grouping similar ideas and combining or removing duplicates. The first author (BW) then amalgamated and summarised the content from both workshops and drafted the initial paper outline, capturing key emerging themes, and distributed this to all workshop participants for feedback and refinement. While the discussions and outputs from the workshop captured a wide range of insights about lived experience inclusion from diverse perspectives, it was agreed to focus the current synthesis on the aspects that were most pertinent to the research community.

### Lessons learned from CCM for lived experience inclusion

Lessons learned are described below with the aim of supporting future lived experience engagement projects, and are organized into the following categories: (a) lessons for all audiences (i.e., any kind of stakeholder engaged in lived experience work), (b) lessons for researchers, (c) lessons for funders, and (d) lessons for policymakers and practitioners. The second set of findings relates to a wider overview of successes, opportunities for future improvement, and the sustainability of the lived experience work.

### Lessons learned for all audiences

Those involved in CCM shared a variety of lessons learned that will benefit any stakeholder audience (i.e. researchers, funders, policymakers, community organizations, etc.) doing future lived experience engagement work. These are organized in Table 1. Below Table 1, the core principles are further described in terms of their specific applicability and salience to researchers, funders, and policymakers/practitioners.

**Table 1:** Synthesis of the overall lessons learned through Lived Experience engagement activities in the Connecting Climate Minds Initiative.

Core Principle	Application in CCM	Illustrative example	Key Challenges	Suggestions for future projects
Relational care and deep listening	Centered lived experience by building trust over time; prioritised flexibility in engagement formats (e.g. in-person and online gatherings with Indigenous communities and young people)	Use of ‘Talanoa’ style dialogues in the Oceania RCC — rooted in Pacific oral traditions, ontologies, and epistemologies [25].	Relational pace slower than typical project timelines; emotional toll on communities and researchers; pushback from funders and academic institutions with specific deadlines.	Build in longer timelines than may seem necessary at first, as well as flexible budgets.
Reciprocity and mutual benefit	Focused on co-design and benefit-sharing; ensured LE advisors could amend or expand their contributions at any time depending on their needs and desired involvement.	LE contributors from SSA shared that their mental health was being impacted by climate-related water insecurity and in response a RCC member provided training in green approaches to water purification. LE contributors were supported to gain access to new platforms and skills (i.e. sent a youth contributor to COP29; several LE contributors supported to attend CCM global event.)	Expectations of benefit sharing were unclear early on; risk of extractive participation; lack of structured processes for sharing benefits equitably.	Clarify the terms of reciprocity and what benefit(s) LE experts want and will get out of participating at the outset of any partnership or engagement.
Diversity, inclusion and avoiding tokenism	Included diverse voices through multiple modalities to increase accessibility (WhatsApp, local gatherings); elevated voices beyond the 'usual suspects' by recruiting through a variety of networks and across countries in each region.	Chose communication systems in partnership with LE team members, prioritised those already used widely and free to access by community, e.g. WhatsApp.	Hard to always identify new voices; risk of LE fatigue; overrepresentation of charismatic or accessible storytellers; researchers faced challenges of prioritising meetings at accessible hours for international contributors.	Strive to have representation from diverse LE communities embedded in the project’s leadership the whole way through, include researchers from disparate regions and timezones, and reflect on ways to include LE voices with less research experience.
Cultural and contextual sensitivity	Flexible, culturally adapted consent processes; adapted mental health language, concepts and framing to match local understandings; supported multilingual facilitation; embraced Indigenous knowledge systems.	According to LE experts’ desires, we incorporated art and visual storytelling as forms of knowledge production and dissemination. Translations were available during dialogues with live translators; participant information sheets and project outputs made accessible in multiple languages.	Varying vocabularies and frameworks for understanding mental health creates confusion and disconnection; requires translation and contextualisation.	Access intercultural communication trainings that are relevant to the areas where work is being done; ensure facilitators are well trained in multiple languages and local mental health context to meet LE needs.
Ethical storytelling and data sovereignty	Prioritised trauma-informed interviewing to gather LE videos; once videos were gathered, they were shared back to sense-check with original contributors; video gatherers trained in Psychological First Aid; upheld consent processes; enabled story co-ownership.	Indigenous data sovereignty frameworks used throughout the project. Contributors of LE videos told exactly how videos would be used and provided consent; would re-consent on any new use cases (e.g. before use at COP28).	Risk of retraumatization; difficult to capture nuance and one’s full truth under time constraints; story oversimplification; overload of time/resource for both researchers and LE communities with a lot of back-and-forth.	Commit to training researchers in LE methods they may not have been previously exposed to; prioritise trauma-informed interviewing; centre story co-ownership and Indigenous data sovereignty.
Long-term engagement and follow-up	Maintained contact and relationships post-project with established LE networks; prioritised continued presence beyond funding window; shared insights and outputs with communities engaged throughout the project.	Fostered connections across regions i.e. Youth Ambassadors were networked with their own Whatsapp group to enable deeper relevant connections. At the CCM global event, the LEWG created check-ins for LE attendees before, during and after the event. LE communities are recontacted when new opportunities arise, e.g. the Lancet Psychiatry Commission.	Time and funding structures misaligned with long-term commitments.	Secure funding necessary for maintaining longer term engagement that has flexible parameters. Must be capable of balancing resource feasibility with genuine appreciation of the slow and at times challenging nature of relationship-based research that depends on cultural humility as well as personal stories and insights.
Openness to power sharing and unlearning	Trained in decolonising research methodologies; integrated Indigenous knowledge, methodologies, and approaches into existing frameworks; prioritised Indigenous sovereignty and data co-ownership; accepted iterative change.	Decolonial training workshop for all CCM team members at the start of the project; worked with institutions to amend research ethics processes to be accommodating of Indigenous sovereignty and data co-ownership frameworks that they had not worked with before (i.e. Imperial College ethics review board eventually accepted collective consent and fingerprints as forms of participant consent).	Institutional resistance to shifting power about how research is done in academia; extra time/budget needed for re-education and trust building.	Prioritize training researchers in decolonising methods up front and maintain consistent training opportunities for ethical engagement; enable feedback sessions on how the process is going and iterate/improve at each step; characteristics of culture change require special funding when budgeting.
Measuring impact of LE engagement	Created outputs including videos, case studies, and co-designed community agendas to show LE power and influence	Release of the CCM Global Online Hub; surveys of LE contributors to gather feedback on their experiences.	Hard to quantify impact; lack of standard metrics for LE engagement .	Think early and innovatively in a project’s life cycle about how to measure the impact of LE engagement.

Legend: Lessons in Table 1 are presented as core principles of LE engagement methods, then described in terms of their application in CCM, with key challenges highlighted that were associated with their application and related suggestions for future projects.

### Lessons for researchers

#### Relational care and deep listening

To become more effective in engaging with lived experience, researchers need to reflect early on how to incorporate lived experience engagement into the fundamental structure of their research, making it an automatic and foundational element rather than an afterthought. Relational care is vital; it is essential to value the individuals involved in the research process as much as the research outputs, even when faced with tight deadlines.

#### Reciprocity and mutual benefit

A key challenge lies in navigating the tension between the aims of the research project and the objectives of lived experience engagement, ensuring that there is reciprocity between researchers and lived experience advisors. Communities should not be viewed merely as sources of data; they should be considered active partners in the research process and the research should consider whether there are direct benefits to individuals and communities from their involvement. CCM contributors highlighted that they can share their experience with researchers yet nothing changes with respect to the challenges they face as a community, leading to distrust and burnout. Many researchers, however, lack training in this area, which highlights the importance of recognizing the limits of their knowledge and capabilities when facilitating conversations about lived experience. It is crucial to prioritize the well-being of participants to avoid revictimization, tokenism, and other common pitfalls, knowing that harmful impact is possible even when researchers hold the best intentions. When gathering first-person narratives of lived experience, it is crucial to apply psychological safeguards and value the presence of psychosocial support during interviews with lived experience experts. This can be achieved by ensuring that those conducting the interviews receive training in Psychological First Aid [26] or a similar trauma-informed approach. The goals of these interviews should never be exploitative; instead they should aim to understand and learn from the wisdom of lived experience experts.

#### Diversity, inclusion and avoiding tokenism

As story gatherers, researchers often gravitate towards individuals who present their narratives in an engaging manner—those who are charismatic or easily accessible, often seen in broadcasts, blog posts and social media. However, it is important for researchers to seek out meaningful voices that are harder to reach or less prominent for whatever reason, to not risk perpetuating inequities by continually re-engaging a small handful of lived experience experts. Our approach included engaging a diverse, bilingual group of participants, prioritizing accessibility through various communication platforms (e.g., WhatsApp), as well as designing project processes and outputs with the accessibility needs of individuals with different abilities in mind. Researchers should be prepared to invest more time, demonstrate greater humility, and exercise more patience and curiosity than they might initially anticipate.

#### Cultural and contextual sensitivity

The frameworks and outcome measures used by researchers do not always reflect the communities they seek to represent. When employing these measures, it is essential to ensure that one is deeply listening to and appropriately reflecting individuals’ lived experiences. Accurately depicting the nuances of mental health is particularly challenging. Therefore, researchers must be prepared to listen to unexpected narratives that may not align with their initial framing, which will yield deeper insights and foster meaningful connections within the community. To facilitate this approach, we implemented several key strategies, including a subgranting model for the LEWG that provided the necessary funding, resources, and infrastructure for local integration. This included, for example, the Indigenous Advisory Board working with the Waman Wasi community leadership to engage Indigenous community members in the Peruvian Amazon. We prioritized culturally sensitive and justice-oriented approaches to language by ensuring that translators were available during the regional dialogues and that participant information sheets were accessible in multiple languages. Additionally, we established feedback mechanisms that allowed lived experience advisors to consent to and amend their contributions at any time.

#### Ethical storytelling and data sovereignty

Simply adhering to standard protocols, such as those outlined in commonly used research ethics documents from universities, may not suffice. Instead, these issues must be discussed explicitly and early with participants, particularly in accordance with Indigenous data sovereignty frameworks [27, 28, 29]. By prioritizing open dialogue about data ownership, researchers can foster a more respectful and equitable relationship with communities involved in research.

#### Openness to power sharing and unlearning

Part of lived experience work involves diffusing the power dynamics between researchers and participants, recognizing that everyone possesses a form of experience that makes them an expert in their own life, including the researchers themselves. This acknowledgment also highlights the importance of caring for the well-being of researchers, as their emotional health is also critical to the research process. Researchers are at risk of experiencing vicarious trauma, compassion fatigue, and overwhelm, which underscores the need for support and self-care in their work.

### Lessons for funders

#### Relational care and deep listening

Overall, a small lived experience budget should be a red flag for funders and researchers alike. Healthy and flexible lived experience budget requests should be welcomed as a sign of commitment rather than questioned. Funders need to stay flexible and responsive throughout the process, understanding that co-produced work cannot always predict exact timelines or costs at the outset. This calls for a mindshift in the funding landscape, where lived experience engagement is centrally valued and understood as being about trust, context, shared ownership and evolving co-created outputs. The goal should not be to fastidiously meet predefined goals and cross items off a checklist.

#### Reciprocity and mutual benefit

A key insight for funders is to consider funding activities that directly benefit lived experience experts involved in the project. This might be trainings, conference participation, or other varied supports. For example, if the research reveals that the community is suffering from a lack of particular protection, knowledge, or skill, could some budget be put towards mitigating that, to ensure that there is a material change in the lives of participants as a result of engaging deeply with the project?

It may also be beneficial for funders to support the establishment of an internal LEWG within any grantee’s project, where that team becomes responsible for bringing individuals with lived experience into the project, collecting data as well as conducting and evaluating lived experience aspects of their programs in a trustworthy and respectful fashion. This team should ideally partner with local groups and interpreters, ensuring equitable distribution of funds is practiced so that marginalized voices are included and able to access opportunities at every step. Once again, having representation on the research and funding teams that reflects the lived experience communities engaged can significantly support this outcome, as can engaging with lived experience advisors throughout a project.

#### Cultural and contextual sensitivity

At times, funders’ lack of understanding around lived experience engagement may be a barrier to a project’s eventual success. True lived experience engagement requires funders to appreciate its sensitivity and depth, and to provide the structures and resources needed to do it well. When a funder has an internal team on staff that is dedicated to lived experience work full-time, as was the case with CCM’s funder (Wellcome Trust), this is a tremendous benefit to any funded lived experience project. But even then, concepts should be held lightly despite a lot of lived experience expertise being present on the funder’s team, as each project has unique histories, inputs, and power dynamics that invite humility and an openness to learning what will be needed for lived experience engagement to be successful. No funder can hit the ground running with pre-formulated ideas about lived experience work; one always needs to make time for context and nuance concerning relationships and historical power imbalances.

#### Long-term engagement and follow-up

Projects also require flexible budgets that have the elastic capacity to stretch over time, as lived experience needs emerge. For instance, there was a strong desire from young people for in-person dialogues to reach people in low digital access zones, which was not known at the outset. Funders can help the success of lived experience engagement by providing support for ongoing capacity building and longer-term relationships, including dissemination of project outcomes in accessible ways back to the communities involved after the initial project deadlines.

### Lessons for policymakers/practitioners

#### Relational care and deep listening

Much as in the case of funders, policymakers and practitioners must prioritize genuine lived experience community engagement by actively incorporating community input into policy decisions as well as building and maintaining transparency about how that input influences outcomes. This helps to prevent “consultation fatigue” and builds long-term trust.

#### Cultural and contextual sensitivity

A one-size-fits-all approach is ineffective for diverse communities at large. Recognizing local needs and asking culturally and contextually rooted people for guidance is key, as is making policies that are built on local evidence. This may require governments to invest in relevant local research and inform researchers about evidence gaps. Throughout CCM, policymakers attended the dialogues to inform the prioritization of research questions being developed in each region.

#### Long-term engagement and follow-up

There should be continuous engagement with policymakers and practitioners, before, during, and after a project, demonstrating commitment to the reciprocal relationship that needs to happen between lived experience-centered research and policy. This is because evidence must inform the policy, and policy needs to be evaluated for its impact on people, including those with lived experience of mental health challenges in the context of climate policy. In addition, policy must be understood as dynamic and open to revision, rather than fixed and untouchable. Post-project engagement is critical because the end of a project does not signify the end of the work.

#### Openness to power sharing and unlearning

Effective policy development should centre the lived experiences of the communities affected, acknowledging that solutions lie within the knowledge communities hold about the challenges they face. Practitioners must remain open to being led by lived experiences, advocating actively, and challenging rigid or outdated policy frameworks that are not equitable. Additionally, policymakers must be prepared to challenge outdated legal structures and colonial norms that centralise knowledge and power in Western academic institutions. A flexible, equity-oriented approach, especially in areas like data co-ownership and Indigenous sovereignty, is crucial [30]. The significant role of policy in decolonizing research and practice includes educating stakeholders and adapting processes to reflect just, inclusive principles.

### Selected examples of how CCM put these learnings into practice

CCM’s LEWG tried to ensure that community members were not treated as mere data sources by providing trainings that covered critical awareness of the ethical and practical considerations one needs to make when engaging with community members to the Global Team, regional community teams, and leaders of the thematic workshops throughout the project. We also shared all project outputs back to community members via the CCM Global Online Hub [5], providing free and full access to the research contents. This established a bidirectional flow of data, including regional and global research and action agendas, toolkits, sub thematic dialog agendas, videos of lived experience stories and project news, between communities and researchers. An example of how community members engaged with the data points towards The Eco-anxiety Africa Project (TEAP https://teap.sustyvibes.org/), which featured the work of CCM and several project outputs in a documentary they produced and use to engage wider communities with lived experience of the mental health impacts of climate change around the world.

Furthermore, CCM made efforts to support community members with material resources, for instance, by sponsoring individuals to attend the UN COP climate negotiations in Azerbaijan and the CCM Global Event in Barbados — important global impact spaces in which to contribute their voices and community perspectives that they otherwise would not have been able to access.

### Successes

Lived experience engagement was recognized by all team members as critical to each aspect of CCM and thoroughly integrated into all levels of the project. The deliberate development of LEWGs, LEAGs, and lived experience community work streams were standout successes. Ceding control to communities to organize their own dialogues according to their preferred cultural protocols, formats, and languages supported diverse forms of knowledge generation and was instrumental for trust-building within the project. For example, by listening to lived experience experts’ desires, we successfully incorporated art and visual storytelling as forms of knowledge production and dissemination. Successes can be seen at all levels of the project outputs that are found on the CCM Global Online Hub [5], where for instance, all regional and global agendas reflect emergent needs of people with lived experience that have not yet been documented or reflected in peer-reviewed research.

By working closely with lived experience advisors, we were able to seed and develop equitable processes within the project’s host university – Imperial College – which the institution had not used before (i.e. Indigenous sovereignty and data co-ownership) [29, 31]. This meant operationalizing decolonial ways of carrying out research ethics and informed consent that Western institutions are not typically accustomed to (i.e. collective consent, use of fingerprints). This introduced new ways of working for the Imperial College Research Ethics Committee and those involved in carrying out the project, demanding patience, openness, humility and goodwill on the part of all actors involved.

Furthermore, CCM nurtured a global audience of people who are interested in learning more about the intersection between climate change and mental health, directly engaging > 1180 participants from 126 countries throughout the project and reaching countless others through its various research, policy, and communications outputs (i.e. media, academic papers, newsletters, events, and the global online hub see: https://hub.connectingclimateminds.org).

While achieving policy changes was not within scope of CCM as a primary project aim, policy changes have been observed. For instance, in Jordan, the CCM team worked with members of the Jordanian government to support the integration of climate change into mental health policies, and mental health into climate change policies. At international levels, the results of CCM have been presented at successive UN Framework Convention on Climate Change conferences, and CCM was a mental health partner that helped develop the Belém Health Action Plan — a global framework launched during the UN Climate Change Conference (COP30) in Belém, Brazil, to place health and equity at the center of climate action [32]. The UK Health Security Agency report on climate change and mental health explicitly positions its report against the CCM agenda, stating that it addresses several research priorities identified in the CCM Global Research and Action Agenda [33]. The National Institute for Health and Care Research in the UK (NIHR) commissioned the “New Perspectives” Centre for Mental Health Review, in which CCM was featured as a case study, with a focus on the lived experience lessons learned. Furthermore, following CCM, the international non-profit United for Global Mental Health established a dedicated LEAG on climate change and mental health, with 12 members spanning six WHO regional groupings, explicitly to address the underrepresentation of people with lived experience in climate and mental health policy advocacy. CCM supported the development of this lived experience group to ensure the lessons learned in CCM inform their ongoing work. Similarly, CCM outputs also led to advocacy that resulted in the Lancet Psychiatry Commission on Climate Change and Mental Health [34], including Commissioners with lived experience of the mental health impacts of climate change from around the world.

We also documented improvements in the conditions of those living with the effects of climate change. A pertinent example comes from the work that was done via CCM representation in Nigeria. There, community members highlighted that water insecurity in the face of floods and droughts was compounding and affecting their mental health in negative ways. A CCM team member ran workshops with the community using CCM resources that integrate Indigenous and scientific knowledge to raise awareness of climate-related distress and how to manage it with support. The workshops also demonstrated practical solutions, including how to use green technologies for water filtration, teaching community members how to generate clean sources of drinking water without intensive environmental impact, which simultaneously explored how such actions can reduce climate-related distress.

### Opportunities for future improvement

Future lived experience engagements can improve upon the practices and procedures outlined in this paper to ensure full cycle participation and leadership of people with lived experiences. While we were able to provide some introductory capacity-building for both lived experience participants and researchers, it was limited to basic content and guidance. We did not reach deeper levels of capacity-building, such as training community leaders to engage effectively with researchers, and vice versa, due to common limitations that flow downstream of project budgets and bandwidth. We see an opportunity for more robust training procedures, as well as qualitative learning approaches and participatory action research methods that would better allow for local adaptations and cultural considerations to be integrated. Furthermore, our initial training on topics that support lived experience work, such as engaging young people and decolonial research methods, was well received and an area that can be deepened in future efforts.

Despite securing small honorariums and payment structures for lived experience contributors, CCM would have benefited from a multi-year funding pathway for lived experience work. With tight deadline cycles over a project’s life, it can be hard to infuse the full wisdom and trust of lived experience contributors into a project, resulting in engagement challenges that could possibly be avoided with a longer runway for the work.

Finally, we observed that approaching the intersection of climate change and mental health from a lived experience perspective is largely unexplored in some regions, presenting a unique opportunity for future research to grow this field of work.

### Sustaining lived experience work moving forward

Maintaining ongoing relationships is central to sustaining lived experience work. By building a robust network that extends beyond the time frame of the project, stakeholders can demonstrate and act upon a continued commitment to lived experience communities in the interest of climate and epistemic justice. Valuing creative modes of dissemination such as storytelling, art, and ceremony, we honour diverse knowledge systems. All too often, research is only disseminated in academic journals with little to no readership within the affected communities, hindering capacity for an informed response to the topic under study.

## Conclusions

Throughout the course of CCM, lived experience engagement was integrated across all working group processes and research outcomes. The lessons learned from these efforts, as reported in this paper, revealed the immense benefit of working with lived experience communities. They also revealed the many systemic barriers which lived experience communities face when engaging in research, which researchers, funders, policymakers/practitioners and other stakeholders must demonstrate care for, in order to pursue meaningful partnerships with those who have first hand experience of the mental health challenges that stem from climate change. For without such partnerships, the most relevant opportunities for change and localized solutions for people and the planet might be entirely missed.

## Data Availability

No datasets were generated or analysed during the current study.

## Abbreviations

CCM: Connecting Climate Minds
LE: lived experience
RCC: regional community convenor
YA: youth ambassadors
LEAG: lived experience advisory group
LEWG: lived experience working group
